# Development of the FAST‐M maternal sepsis bundle for use in low‐resource settings: a modified Delphi process

**DOI:** 10.1111/1471-0528.16005

**Published:** 2019-12-05

**Authors:** D Lissauer, J Cheshire, C Dunlop, F Taki, A Wilson, JM Smith, R Daniels, N Kissoon, A Malata, T Chirwa, VM Lwesha, C Mhango, E Mhango, C Makwenda, L Banda, L Munthali, B Nambiar, J Hussein, HM Williams, AJ Devall, I Gallos, A Merriel, M Bonet, JP Souza, A Coomarasamy

**Affiliations:** ^1^ Institute of Translational Medicine University of Liverpool Liverpool UK; ^2^ Malawi‐Liverpool‐Wellcome Trust Clinical Research Programme College of Medicine Blantyre Malawi; ^3^ Institute of Metabolism and Systems Research University of Birmingham Birmingham UK; ^4^ World Health Organization Collaborating Centre for Global Women’s Health Research University of Birmingham Birmingham UK; ^5^ The Hillingdon Hospitals NHS Foundation Trust London UK; ^6^ Institute of Applied Health Research University of Birmingham Birmingham UK; ^7^ The Bill & Melinda Gates Foundation’s Maternal, Newborn & Child Health Team Seattle WA USA; ^8^ University Hospitals Birmingham NHS Foundation Trust Birmingham UK; ^9^ Department of Pediatrics and Emergency Medicine University of British Columbia Vancouver BC Canada; ^10^ Malawi University of Science and Technology Limbe Malawi; ^11^ Chitipa District Hospital Chitipa Malawi; ^12^ Save the Children Norway Lilongwe Malawi; ^13^ College of Medicine University of Malawi Blantyre Malawi; ^14^ Parent and Child Health Initiative (PACHI) Trust Lilongwe Malawi; ^15^ Institute for Global Child Health University College London London UK; ^16^ Independent Maternal Health Consultant Aberdeen UK; ^17^ Institute of Clinical Sciences College of Medical and Dental Sciences University of Birmingham Birmingham UK; ^18^ School of Population Health Sciences Bristol Medical School University of Bristol Bristol UK; ^19^ UNDP/UNFPA/UNICEF/WHO/World Bank Special Programme of Research, Development and Research Training in Human Reproduction (HRP) Department of Reproductive Health and Research World Health Organization Geneva Switzerland; ^20^ Department of Social Medicine Ribeirão Preto Medical School University of Sao Paulo Ribeirão Preto Brazil

**Keywords:** care bundle, Delphi process, low resource setting, maternal sepsis

## Abstract

**Objective:**

To develop a sepsis care bundle for the initial management of maternal sepsis in low resource settings.

**Design:**

Modified Delphi process.

**Setting:**

Participants from 34 countries.

**Population:**

Healthcare practitioners working in low resource settings (*n* = 143; 34 countries), members of an expert panel (*n* = 11) and consultation with the World Health Organization Global Maternal and Neonatal Sepsis Initiative technical working group.

**Methods:**

We reviewed the literature to identify all potential interventions and practices around the initial management of sepsis that could be bundled together. A modified Delphi process, using an online questionnaire and in‐person meetings, was then undertaken to gain consensus on bundle items. Participants ranked potential bundle items in terms of perceived importance and feasibility, considering their use in both hospitals and health centres. Findings from the healthcare practitioners were then triangulated with those of the experts.

**Main outcome measure:**

Consensus on bundle items.

**Results:**

Consensus was reached after three consultation rounds, with the same items deemed most important and feasible by both the healthcare practitioners and expert panel. Final bundle items selected were: (1) Fluids, (2) Antibiotics, (3) Source identification and control, (4) Transfer (to appropriate higher‐level care) and (5) Monitoring (of both mother and neonate as appropriate). The bundle was given the acronym ‘FAST‐M’.

**Conclusion:**

A clinically relevant maternal sepsis bundle for low resource settings has been developed by international consensus.

**Tweetable abstract:**

A maternal sepsis bundle for low resource settings has been developed by international consensus.

## Tweetable abstract

A maternal sepsis bundle for low resource settings has been developed by international consensus.

## Introduction

Maternal sepsis is defined as ‘organ dysfunction resulting from infection during pregnancy, childbirth, post‐abortion, or the post‐partum period’.^1^ It is the third most common direct cause of maternal mortality, accounting for 11% of maternal deaths worldwide and disproportionately affecting low‐ and middle‐income countries (LMICs).^2^ In 2013, the WHO estimated that 99% of the 302 000 maternal deaths had occurred in LMICs.^3^

Until now, attempts to address Sustainable Development Goal 3.1 of *reducing global maternal mortality ratio to less than 70 per 100 000 live births* 4 have often focused on haemorrhage and hypertensive disorders, with maternal sepsis receiving less attention.^5^

The World Health Assembly has recognised sepsis as a global health priority and has adopted a resolution to improve the prevention, detection and management of sepsis.^6^ As part of the effort to address this and in recognition that only modest inroads have been made in decreasing the burden and poor outcomes of maternal and neonatal sepsis, the World Health Organization (WHO) and Jhpiego launched the ‘Global Maternal and Neonatal Sepsis Initiative’ with an objective to *develop and test effective strategies to prevent, detect and successfully manage maternal and neonatal sepsis*. Development of an internationally recognised maternal sepsis bundle is a key step in this strategy.^5^

Care bundles are *a set of evidence‐based practices, generally three to five items, that when performed collectively and reliably, improve patient outcomes*.^7^ Compliance with a bundled approach to sepsis management has been demonstrated to reduce mortality,^8^, ^9^ and have been a cornerstone of sepsis improvement initiatives in high‐income settings.^8^, ^9^, ^10^, ^11^, ^12^ Most notably, implementation of the Surviving Sepsis Campaign’s bundle has been associated with a reduction in mortality.^12^, ^13^ Despite this, there are no internationally recognised sepsis care bundles that are specific to the maternal population and can be implemented in a low‐resource setting.^14^, ^15^, ^16^ A continent‐wide survey in Africa^15^ revealed that only 1.5% (*n* = 4/263) of healthcare facilities surveyed could implement the SSC guidelines in their entirety. The inability reliably to implement recommended sepsis management strategies within a low‐resource setting highlights the need for a new approach. The development of a maternal sepsis bundle for low‐resource settings has been identified as an international ‘Priority Action’.^5^, ^17^

Our work aimed to carry out this directive by developing a maternal sepsis bundle for the initial management of maternal sepsis in low resource settings.

## Methods

### Selection of items to include in the Delphi

A comprehensive list of potential treatment and monitoring items was obtained for consideration in the consensus process. National and international guidelines, as well as relevant articles, were reviewed until saturation was achieved (Appendix S1). We screened 217 items for potential inclusion, removed duplicates and grouped similar items. A list of 41 items was then shared with an advisory panel of experts in global health and sepsis comprising doctors and academics working in the fields of obstetrics, neonatology, and anaesthetics. Three items – immunoglobulin therapy, vasopressors and central venous monitoring – were deemed too infeasible to implement in a low resource and were excluded prior to the Delphi process. A final list of 38 items was developed into an online questionnaire (Appendix S2).

### Modified Delphi process

To develop a care bundle for the initial management of maternal sepsis in low resource settings, we sought to form a consensus by eliciting the views of both healthcare practitioners and experts within maternal and perinatal care.

**Figure 1:**
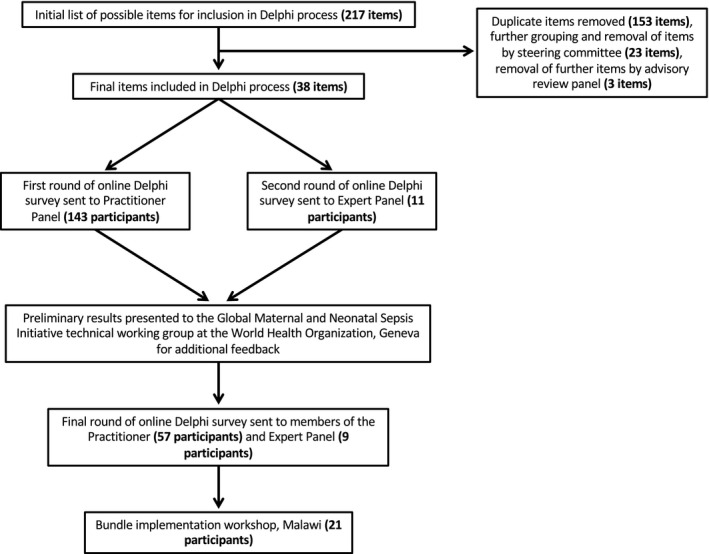
Flowchart depicting the modified Delphi process used to derive the treatment and monitoring components of the FAST‐M maternal sepsis bundle.

The consensus process incorporated a modified three‐step Delphi process,^18^ which took place between December 2015 and May 2016. Figure 1 illustrates the modified Delphi process.

During the first round, an online questionnaire was distributed to healthcare practitioners only. Eligible participants were either those currently working in LMICs or those who had prior experience of working with mothers with sepsis in LMICs. Countries were considered to be low resource if they met the World Bank Classification^19^ of low‐ or lower‐middle‐income countries. Suitable healthcare practitioners were identified through professional networks, literature reviews, and online searches, and were recruited by email from a range of healthcare settings.

In the second round, the same questionnaire was distributed to the expert panel to triangulate against the responses from the healthcare practitioners. The expert group consisted of international experts within the field of global health, sepsis, and obstetrics. An effort was made to ensure that a range of expertise was included. Group members were contacted by email and invited to take part in the same manner as the healthcare practitioners. Results from round one were not revealed to the expert panel.

Participants who consented to be involved were sent a link to an online questionnaire (Appendix S2) to complete. The questionnaire was in English. Initial screening questions ascertained the participant’s role and clinical experience of managing sepsis. Participants were asked to rate potential bundle items, which included both treatment and monitoring components. Likert scales were used to score all potential items in terms both of their importance in the immediate management of maternal sepsis (within 3 hours) and feasibility to be adopted both within a health centre and hospital setting of a low‐or lower‐middle‐income country. Questions relating to importance were scored 1–5 on the Likert scale, with ‘1’ being very un‐important and ‘5’ being very important. Questions relating to feasibility were scored 1–4, where ‘1’ was definitely un‐feasible and ‘4’ was definitely feasible. Spaces for free text were included to enable participants to suggest any additional items they felt should be included. No incentive was offered for completing the questionnaire.

Following the second round, preliminary results from the Delphi process were presented to the WHO Global Maternal and Neonatal Sepsis Initiative technical working group. This was not a formal round of the Delphi process but was an additional opportunity to gather expert opinion and seek suggestions.

In the final round, the results of the responses from the first two rounds were re‐distributed to both the healthcare practitioners and expert panel, along with a final list of bundle items. The final list of treatment items presented was limited to five items, in line with the recommended number of components of a care bundle.^7^ An online questionnaire (Appendix S3) sought to determine consensus on these finalised bundle items.

Following completion of the modified Delphi process, a bundle implementation workshop, attended by policymakers and practitioners (21 participants), was conducted in Lilongwe, Malawi. This site was chosen as it was planned as the site for future feasibility testing. The purpose of this Bundle implementation workshop was to conclude the consensus‐forming process with a wide range of stakeholders and to discuss methods for the implementation and evaluation of the maternal sepsis bundle. The final components of the bundle were presented and further feedback sought.

### Role of the funding source

Research funding was provided by University of Birmingham and the charity Ammalife. Several authors are, or have recently been, employed by the University of Birmingham. D.L., A.C., J.C., and C.D. all work with the charity Ammalife as volunteers. Those engaged in the work were excluded from the funding decision‐making by Ammalife. Neither funder had input into the study design, data collection, data analysis, data interpretation or writing of the report.

## Results

Overall 154 participants from 34 LMICs (Africa: 92/143 [64%], Asia: 22/143 [15%], Europe: 17/143 [12%], South America: 11/143 [8%] and North America: 1/143 [1%]) participated in the Delphi process. The practitioner panel included 143 members from speciality 81/143 (57%) and non‐speciality 7/143 (5%) physicians, midwives, and nurses 12/143 (8%), non‐physician clinicians 27/143 (19%), clinical scientists 6/143 (4%) and ‘other’ 10/143 (7%), working in a range of settings (tertiary care: 64/143 [45%], district hospital: 56/143 [39%], health centres: 12/143 [8%] and other: 11/143 [8%]). When asked about their experience in managing maternal sepsis, 110/143 (77%) responded that they had managed a case of maternal sepsis in the preceding 6 months. The expert panel consisted of 11 members, including six obstetricians, one paediatric intensivist, one infectious disease physician, and one emergency medicine physician; two members were senior global maternal health academics. All members of the expert panel had experience in implementing international health quality improvement projects in the fields of sepsis and maternal health.

**Figure 2:**
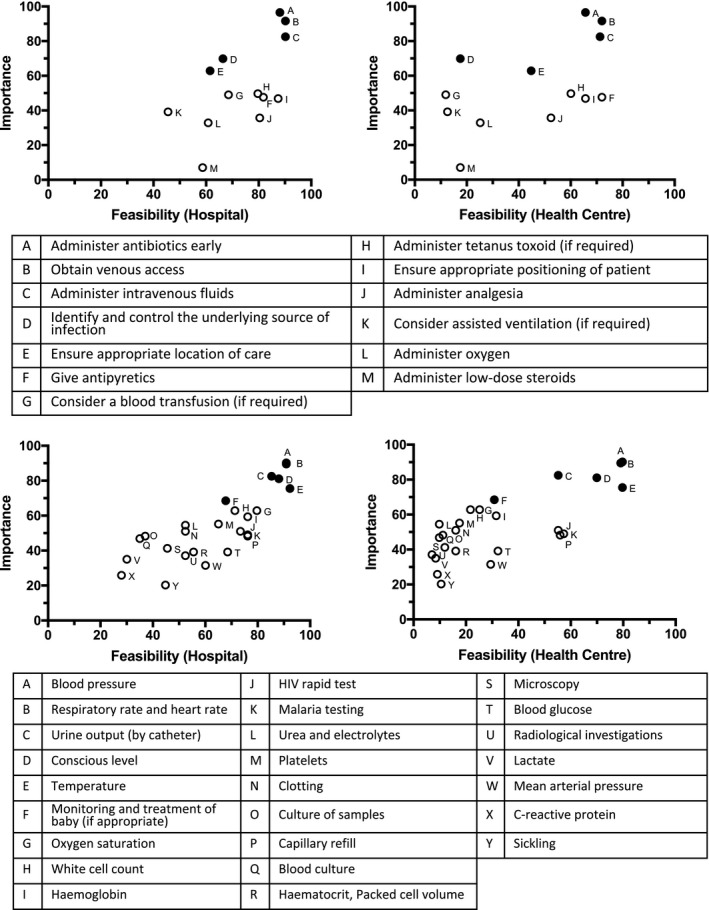
Perceived importance and feasibility of treatment and monitoring items in both a hospital and health centre setting.

The first round of the Delphi process ranked potential bundle items by clinical importance and implementation feasibility, in both a hospital and health centre setting (Figure 2A–D). The treatment item perceived to be most important by the highest number of respondents was early administration of antibiotics; 138/143 (97%) of healthcare practitioners rated it ‘very important’. Additional items frequently ranked as important or very important included: obtaining venous access 131/143 (92%), administering intravenous fluid 118/143 (83%), identifying and treating the underlying source of infection 100/143 (70%), and ensuring appropriate location for care 90/143 (63%) (Figure S1). In terms of feasibility, the following treatment items were deemed the most feasible to implement in a health centre setting; antipyretics 103/143 (72%), obtaining venous access 103/143 (72%), administration of intravenous fluids 102/143 (71%), early administration of antibiotics 94/143 (66%), and correct positioning of the patient 94/143 (66%) (Figure S2). The overall feasibility of implementing these items was higher in the hospital setting (Figure S3).

The monitoring items perceived to be most important by the highest number of respondents were measurement of the patient’s blood pressure 129/143 (90%) along with measurement of the patient’s respiratory rate and heart rate 128/143 (90%). Additional items frequently ranked as very important included: measurement of urine output by catheter,118/143 (83%); assessment of consciousness level, 116/143 (81%); measurement of temperature, 108/143 (76%), and monitoring of the newborn, if applicable, 99/143 (69%) (Figure S4). Monitoring items deemed most feasible to implement in a health centre setting were measurement of blood pressure, 114/143 (80%); measurement of temperature, 114/143 (80%); measurement of respiratory rate and heart rate, 113/143 (79%); assessment of consciousness level, 100/143 (70%); malaria testing, 82/143 (57%); measurement of capillary refill, 80/143 (56%); rapid HIV test, 79/143 (55%); and measurement of urine output by catheter, 79/143 (55%) (Figure S5). Similarly, in a hospital setting these items were also deemed most feasible, although measurement of urine output by catheter was perceived to be more feasible than in the health centre setting (122/143, 85% versus 79/143, 55%) (Figure S6).

The second round of the Delphi process asked the expert panel to rate the importance of potential bundle items, in both a hospital and health centre setting (Figures S7 and S8). The results of the expert panel were consistent with those of the practitioner panel, rating the same components as being the most important in the immediate treatment of maternal sepsis and in the initial monitoring of women with maternal sepsis (Table 1).

**Table 1:** Most important treatment and monitoring items as ranked by the practitioner and expert panels according to their mean score on the 5‐point Likert scale

Practitioner panel	Rank	Expert panel
**Treatment items**		
Antibiotics Mean = 4.90, SD = 0.58	1	Antibiotics Mean = 5.00, SD = 0.00
Intravenous access Mean = 4.83, SD = 0.69	2	Intravenous access Mean = 5.00, SD = 0.00
Intravenous fluids Mean = 4.71, SD = 0.76	3	Intravenous fluids Mean = 4.64, SD = 0.48
Source identification and control Mean = 4.56, SD = 0.82	4	Location Mean = 4.36, SD = 0.64
Location Mean = 4.39, SD = 0.97	5	Source identification and control Mean = 4.09, SD = 1.16
**Monitoring items**		
Blood pressure Mean = 4.85, SD = 0.58	1	Respiratory rate and heart rate Mean = 4.82, SD = 0.39
Respiratory rate and heart rate Mean = 4.80, SD = 0.70	2	Conscious level Mean = 4.82, SD = 0.39
Urine output Mean = 4.73, SD = 0.69	3	Urine output Mean = 4.64, SD = 0.48
Conscious level Mean = 4.71, SD = 0.71	4	Monitoring of baby Mean = 4.64, SD = 0.48
Temperature Mean = 4.65, SD = 0.75	5	Blood pressure Mean = 4.55, SD = 0.66
Monitoring of baby Mean = 4.46, SD = 0.94	6	Temperature Mean = 4.27, SD = 0.96
Oxygen saturations Mean = 4.38, SD = 1.00	7	Oxygen saturations Mean = 4.27, SD = 0.96

SD, standard deviation.

Observations made at the WHO Global Maternal and Neonatal Sepsis Initiative technical working group consultation, after reviewing the first two rounds of the Delphi process, were that resource availability varied widely among healthcare facilities within low‐resource settings and the proposed bundle should be implementable across the varied settings. Furthermore, it was noted that although the assumption had been made that the measurement of oxygen saturations was not sufficiently feasible to warrant inclusion in a bundle, consideration must be given to the fact that in some healthcare settings it will be available.

All participants from rounds one and two were invited to take part in the third round of the Delphi process. Of these, a total of 66 participants participated: 57 of the healthcare practitioners and nine members of the expert panel. Good agreement was achieved with all but two healthcare practitioners, with 55/57 (97%) agreeing with the final treatment components and 56/57 (98%) of healthcare practitioners agreeing with the final selection of monitoring components for inclusion. All members of the expert panel agreed with the final treatment and monitoring components.

Based on this Delphi process, the final bundle was developed to incorporate the following items: Fluids, Antibiotics, Source identification and control, Transfer to an appropriate level of care, and ongoing Monitoring of mother and neonate. Intravenous access was removed from the final bundle, as it was a prerequisite to administering intravenous antibiotics and fluids rather than an individual key component. To aid practitioner recall, the bundle was given the acronym ‘FAST‐M’. The seven monitoring items agreed upon in the Delphi process constituted the monitoring component of the FAST‐M bundle.

The results of the Delphi process were presented at the bundle implementation workshop, at which further agreement on the FAST‐M bundle items was achieved.

## Discussion

### Main findings

An evidence‐informed and consensus‐driven modified Delphi process was conducted to develop the first maternity‐specific sepsis care bundle for use in low‐resource settings. The final items included in the bundle were fluids, antibiotics, source identification and control, transport and monitoring. This has been abbreviated to the acronym FAST‐M for ease of practitioner recall.

### Strengths and limitations

Strengths of our approach include that we derived consensus from a large number of healthcare practitioners and subject experts, who were familiar with working in a range of low‐resource settings. This included representation from Africa, South America, and Asia. Triangulating the responses of the healthcare practitioners with that of an expert panel enabled us to validate the findings between these groups. Despite the range of perspectives, consensus was rapidly reached within three rounds, with high levels of agreement within both the expert panel and the healthcare practitioner panel.

Identification of the majority of the healthcare practitioner panel relied upon stakeholders cascading the invitation to fellow eligible colleagues and therefore may not be representative of all practitioners in that setting. In particular, most practitioners were employed by larger health facilities. Additionally we have not defined what the neonatal monitoring and subsequent treatment should be; however, work is underway to address this separately.

### Interpretation

In our Delphi, lactate, blood cultures and oxygen therapy were mostly perceived as being of reasonable importance; however, the feasibility of reliably implementing these items in low‐resource settings limited their usefulness as bundle elements. This is consistent with the reported scarcity of the required resources to measure lactate, test blood cultures or reliably deliver oxygen therapy in resource‐poor settings.^16^

Although monitoring a patient’s oxygen saturations was perceived as very important by both the healthcare practitioner and expert panel, the inability of many facilities in low‐resource settings to measure oxygen saturations means that this item will not be applicable in many settings. Monitoring of oxygen saturations was not considered always feasible, but its monitoring should be advocated in facilities where it is feasible to do so; this advice is consistent with other current recommendations, in which oxygen therapy is not suggested to be a universal requirement for sepsis care,^10^, ^20^ but is recommended to be applied to patients with inadequate oxygen saturation.^21^

There have been previous attempts to develop sepsis care bundles for low‐resource settings, though they are not widely used or optimised for the obstetric population (Table 2). Dünser et al.^22^ collaborated with a global working group in intensive care to develop recommendations for sepsis care in low‐resource settings. Development of two suggested care bundles followed, with an ‘acute care bundle’ comprising oxygen therapy, fluid resuscitation, early and adequate antimicrobial therapy and surgical source control, and a ‘post‐acute care bundle’ consisting of re‐evaluation of antimicrobial therapy, deep vein thrombosis prophylaxis, glucose control and weaning of invasive support.^22^ Our FAST‐M maternal sepsis bundle should be considered an acute phase bundle.

**Table 2:** Comparison of monitoring and treatment items included in the FAST‐M maternal sepsis bundle with existing sepsis bundles

	Surviving Sepsis Campaign’s resuscitation bundle^10^	UK Sepsis Trust’s Sepsis Six bundle^11^	Dünser et al. Acute care bundle^22^	FAST‐M maternal sepsis bundle
Lactate	X	X		
Blood culture	X	X		
Antibiotics	X	X	X	X
Fluids	X	X	X	X
Oxygen		X	X	
Urine output*		X		X
Monitoring of mother				X
Monitoring of neonate				X
Source identification and control			X	X
Transfer to higher level care				X

a Urine output monitoring is a requirement of the monitoring component of the FAST‐M maternal sepsis bundle.

We note that the final components of the FAST‐M bundle are consistent with those of Dünser et al.’s^22^ acute care bundle, except for the exclusion of oxygen therapy, and the inclusion of maternal and fetal or neonatal monitoring. Inclusion of neonatal monitoring in our bundle is unique and important given that maternal infection is a major risk factor for early‐onset neonatal infection.^23^ Maternal illness mandates the need for additional care of the neonate, as critical illness in the mother often contributes to increased morbidity and mortality in the neonate.^24^, ^25^, ^26^

Sepsis strategies for use in low‐resource settings must be simple to implement, considerate of local resources and available personnel. When developing this bundle for low‐resource settings, we considered not only the importance but also the feasibility of implementing each item. To maximise effectiveness, all components of the FAST‐M bundle must be completed promptly in every patient. If any element of the FAST‐M bundle cannot be addressed at a particular facility, the patient should be transferred to an appropriate setting that can complete the remaining bundle components. Use of the FAST‐M bundle, in its intended setting, will need practical and timely triggers. For example, using deranged maternal vital signs rather than relying on evidence of haematological and biochemical derangement, which are unlikely to be available at these facilities. Unlike many approaches in high‐resource settings, a wide range of healthcare practitioners in both a health centre and hospital setting can implement all elements of the FAST‐M bundle. This is particularly important when considering the time‐sensitive nature of sepsis management and lack of critical care facilities.

Having developed a maternal sepsis bundle for low‐resource settings, we recommend that a suitable implementation approach and the required implementation tools be developed. Such an implementation approach should focus on improving healthcare practitioner’s awareness of the time‐critical nature of maternal sepsis in addition to developing tools to improve its early recognition. We further recommend that the effectiveness of the bundle be evaluated in the clinical setting for which it was developed. Although seeking to validate and test the bundle clinically was beyond the scope of this paper, a multicentre feasibility study assessing whether the introduction of the FAST‐M maternal sepsis bundle is possible in a low‐resource setting is currently taking place (ISRCTN 87339737). If deemed feasible, a definitive trial to determine the ability of FAST‐M to reduce maternal mortality and severe morbidity is recommended.

## Conclusion

We describe the process by which a care bundle for the initial management of maternal sepsis in low‐resource settings has been developed using a consensus‐forming modified Delphi process. Engagement of a large number of healthcare practitioners from a wide range of countries, and an expert panel, has ensured the development of a clinically relevant care bundle for the immediate management of maternal sepsis in low‐resource settings.

### Disclosure of interests

Dr Daniels undertakes consultancy work in addition to his voluntary work for the registered charity UK Sepsis Trust. The charity receives public and philanthropic funds. The other authors declare no competing interests. Completed disclosure of interest forms are available to view online as supporting information.

### Contribution to authorship

DL, AC, FT, AW, HMW, and AJD conceived the research project. DL, FT, AW, JMS, RD, NK, AM, TC, VML, CM, EM, CM, LB, LM, BN, JH, AM, MB, JPS, and AC participated in the data collection. FT analysed the data. FT, AW, DL, JC, IG, and CD interpreted the findings. JC, DL, and CD wrote the first draft, revised subsequent drafts, and prepared the manuscript. All authors contributed equally to the revision of the manuscript and approved the final version of the paper. JC coordinated the contributors.

### Details of ethics approval

Ethics approval to undertake this work was granted by the University of Birmingham’s research ethics team (ERN_16‐1168; date of approval 22 October 2015).

### Funding

University of Birmingham (RG_16‐150) and Ammalife (1120236).

## Supporting information


**Figure S1.** Perceived importance of treatment items by healthcare practitioner panel.
**Figure S2.** Perceived feasibility of treatment items in a health centre setting by healthcare practitioner panel.
**Figure S3.** Perceived feasibility of treatment items in a hospital setting by healthcare practitioner panel.
**Figure S4.** Perceived importance of monitoring items by healthcare practitioner panel.
**Figure S5.** Perceived feasibility of monitoring items in a health centre setting by healthcare practitioner panel.
**Figure S6.** Perceived feasibility of monitoring items in a hospital setting by healthcare practitioner panel.
**Figure S7.** Perceived importance of treatment items by expert panel.
**Figure S8.** Perceived importance of monitoring items by expert panel.

**Appendix S1.** List of international guidance, national guidelines and original articles used to identify treatment and monitoring items for inclusion in the modified Delphi process.
**Appendix S2.** Questionnaire used in rounds one and two of the Delphi study.
**Appendix S3.** Questionnaire used in round three of the Delphi study.

## References

[1] Bonet M , Pileggi V , Rijken MJ , Coomarasamy A , Lissauer D , Souza JP , et al. Towards a consensus definition of maternal sepsis: results of a systematic review and expert consultation. Reprod Health 2017;14:67. 28558733 10.1186/s12978-017-0321-6 PMC5450299

[2] Say L , Chou D , Gemmill A , Tunçalp Ö , Moller A‐B , Daniels J , et al. Global causes of maternal death: a WHO systematic analysis. Lancet Glob Health 2014;2:e323–33. 25103301 10.1016/S2214-109X(14)70227-X

[3] World Health Organization . Trends in Maternal Mortality: 1990 to 2015; estimates by WHO, UNICEF, UNFPA, World Bank Group and the United Nations Population Division. Geneva: WHO; 2015.

[4] World Health Organization . SDG 3: Ensure healthy lives and promote wellbeing for all at all ages. Sustainable development goals [Internet]. 2017 [www.who.int/sdg/targets/en/]. Accessed 1 October 2018.

[5] World Health Organization . Statement on Maternal Sepsis. Human Reproduction Programme. Geneva: WHO; 2017.

[6] Reinhart K , Daniels R , Kissoon N , Machado FR , Schachter RD , Finfer S . Recognizing sepsis as a global health priority—a WHO resolution. N Engl J Med 2017;377:414–7. 28658587 10.1056/NEJMp1707170

[7] Institute for Healthcare Improvement . Evidence‐based care bundles [Internet]. [www.ihi.org/Topics/Bundles/Pages/default.aspx]. Accessed 12 February 2019.

[8] Damiani E , Donati A , Serafini G , Rinaldi L , Adrario E , Pelaia P , et al. Effect of performance improvement programs on compliance with sepsis bundles and mortality: a systematic review and meta‐analysis of observational studies. PLoS ONE 2015;10:e0125827. 25946168 10.1371/journal.pone.0125827 PMC4422717

[9] Barochia AV , Cui X , Vitberg D , Suffredini AF , O’Grady NP , Banks SM , et al. Bundled care for septic shock: an analysis of clinical trials. Crit Care Med 2010;38:668–78. 20029343 10.1097/CCM.0b013e3181cb0ddf PMC3383776

[10] Rhodes A , Evans LE , Alhazzani W , Levy MM , Antonelli M , Ferrer R , et al. Surviving Sepsis Campaign: international guidelines for management of sepsis and septic shock: 2016. Intensive Care Med 2017;43:304–77. 28101605 10.1007/s00134-017-4683-6

[11] Robson WP , Daniel R . The sepsis six: helping patients to survive sepsis. Br J Nurs 2008;17:16–21. 18399392 10.12968/bjon.2008.17.Sup1.28145

[12] Levy MM , Dellinger RP , Townsend SR , Linde‐Zwirble WT , Marshall JC , Bion J , et al. The Surviving Sepsis Campaign: results of an international guideline‐based performance improvement program targeting severe sepsis. Crit Care Med 2010;38:367–74. 20035219 10.1097/CCM.0b013e3181cb0cdc

[13] Seymour CW , Gesten F , Prescott HC , Friedrich ME , Iwashyna TJ , Phillips GS , et al. Time to treatment and mortality during mandated emergency care for sepsis. N Engl J Med 2017;376:2235–44. 28528569 10.1056/NEJMoa1703058 PMC5538258

[14] Bataar O , Lundeg G , Tsenddorj G , Jochberger S , Grander W , Baelani I , et al. Nationwide survey on resource availability for implementing current sepsis guidelines in Mongolia. Bull World Health Organ 2010;88:839–46. 21076565 10.2471/BLT.10.077073 PMC2971517

[15] Baelani I , Jochberger S , Laimer T , Otieno D , Kabutu J , Wilson I , et al. Availability of critical care resources to treat patients with severe sepsis or septic shock in Africa: a self‐reported, continent‐wide survey of anaesthesia providers. Crit Care 2011;15:R10. 21219619 10.1186/cc9410 PMC3222039

[16] Abdu M , Wilson A , Mhango C , Taki F , Coomarasamy A , Lissauer D . Resource availability for the management of maternal sepsis in Malawi, other low‐income countries, and lower‐middle‐income countries. Int J Gynecol Obstet 2018;140:175–83. 10.1002/ijgo.12350 29027207

[17] Jhpiego . Enhancing the Focus on Maternal Infection: Report of a Consultative Meeting. Baltimore: Jhpiego; 2015.

[18] Dalkey N , Helmer O . An experimental application of the DELPHI method to the use of experts. Manage Sci 1963;9:458–67.

[19] The World Bank . Data: World Bank Country and Lending Groups [Internet]. [https://datahelpdesk.worldbank.org/knowledgebase/articles/906519-world-bank-country-and-lending-groups]. Accessed 12 February 2019.

[20] Levy MM , Evans LE , Rhodes A . The surviving sepsis campaign bundle: 2018 update. Intensive Care Med 2018;46:997–1000. 10.1097/CCM.0000000000003119 29767636

[21] National Institute for Health and Care Excellence (NICE) . Sepsis: Recognition, Assessment and Early Management. London: NICE; 2016. 27441326

[22] Dünser MW , Festic E , Dondorp A , Kissoon N , Ganbat T , Kwizera A , et al. Recommendations for sepsis management in resource‐limited settings. Intensive Care Med 2012;38:557–74. 22349419 10.1007/s00134-012-2468-5 PMC3307996

[23] National Institute for Health and Care Excellence (NICE) . Neonatal Infection (Early Onset): Antibiotics for Prevention and Treatment (CG149). London: NICE; 2012.

[24] Bhutta ZA , Lassi ZS , Blanc A , Donnay F . Linkages among reproductive health, maternal health, and perinatal outcomes. Semin Perinatol 2010;34:434–45. 21094418 10.1053/j.semperi.2010.09.002

[25] Ronsmans C , Chowdhury ME , Dasgupta SK , Ahmed A , Koblinsky M . Effect of parent’s death on child survival in rural Bangladesh: a cohort study. Lancet 2010;375:2024–31. 20569842 10.1016/S0140-6736(10)60704-0

[26] Anderson FWJ , Morton SU , Naik S , Gebrian B . Maternal mortality and the consequences on infant and child survival in rural Haiti. Matern Child Health J 2007;11:395–401. 17265193 10.1007/s10995-006-0173-0

