# Supplementary material for: Development of the FAST‐M maternal sepsis bundle for use in low‐resource settings: a modified Delphi process
Source: BJOG. 2019 Dec 5;127(3):416–23. doi: 10.1111/1471-0528.16005 (PMC7384197; doi:10.1111/1471-0528.16005)
Supplement: Supplementary file 1 — Figure S1. Perceived importance of treatment items by healthcare practitioner panel. Figure S2. Perceived feasibility of treatment items in a health centre setting by healthcare practitioner panel. Figure S3. Perceived feasibility of treatment items in a hospital setting by healthcare practitioner panel. Figure S4. Perceived importance of monitoring items by healthcare practitioner panel. Figure S5. Perceived feasibility of monitoring items in a health centre setting by healthcare practitioner panel. Figure S6. Perceived feasibility of monitoring items in a hospital setting by healthcare practitioner panel. Figure S7. Perceived importance of treatment items by expert panel. Figure S8. Perceived importance of monitoring items by expert panel. [file BJO-127-416-s001.pdf]

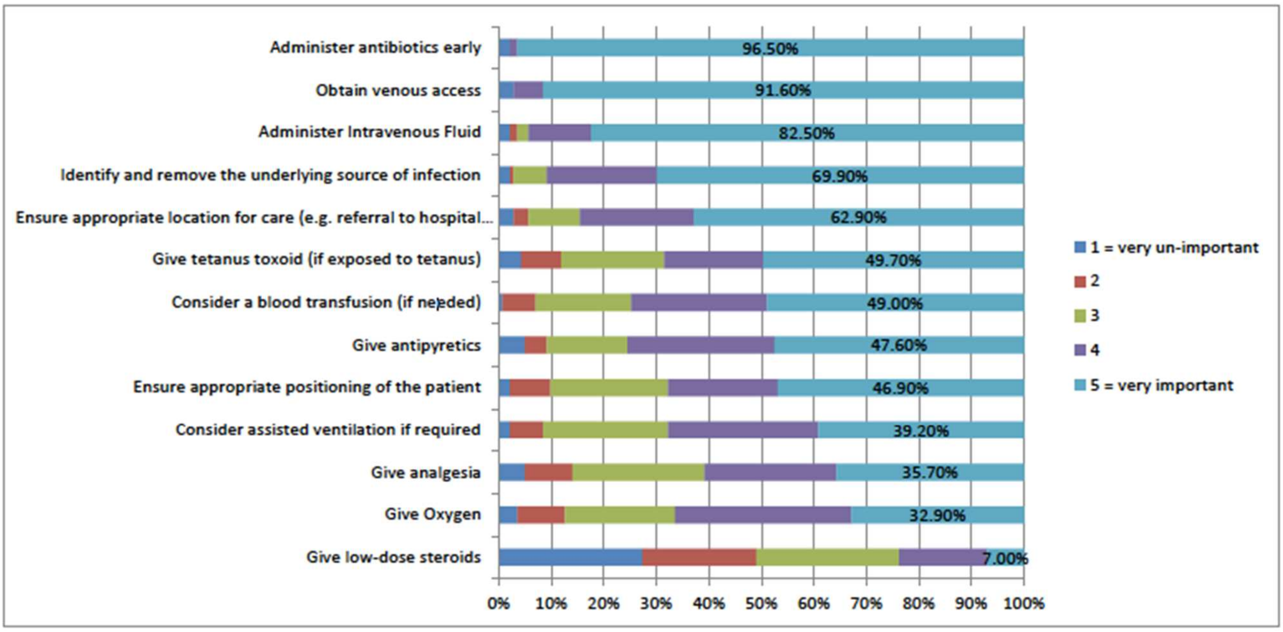

Figure S1. Perceived importance of treatment items by healthcare practitioner panel.

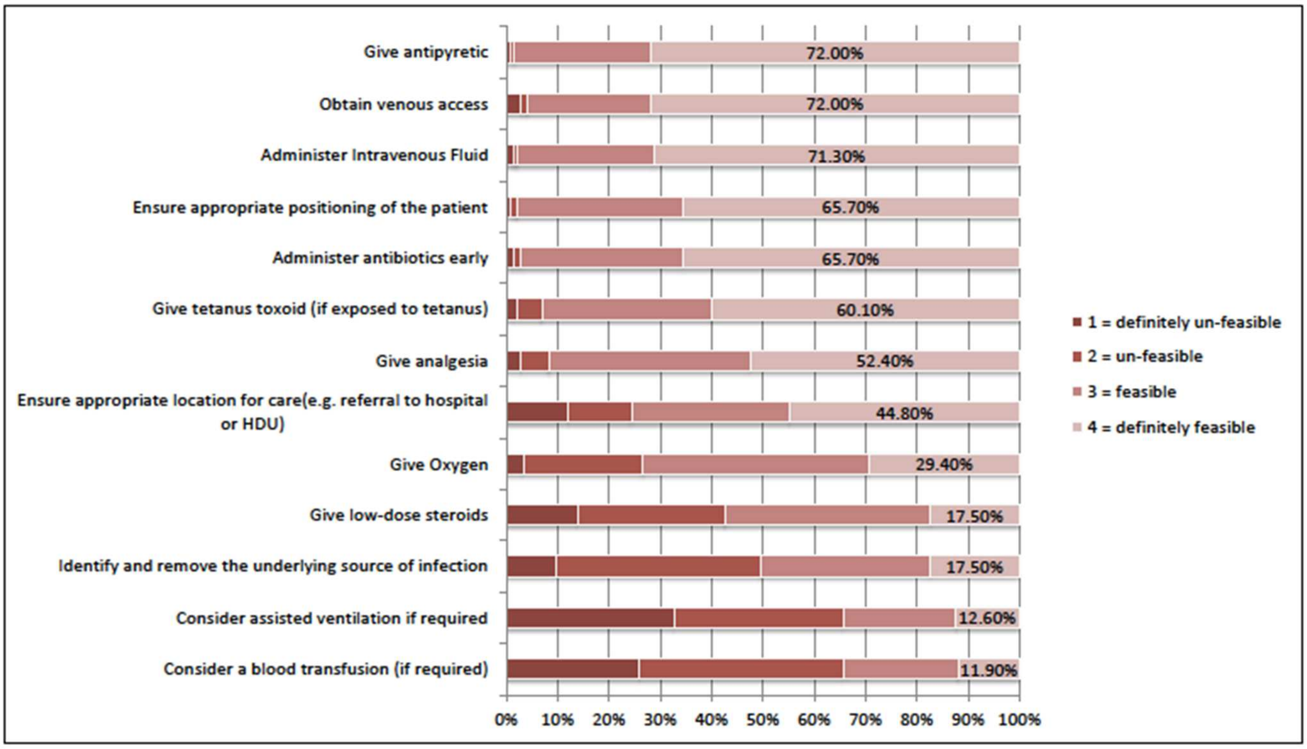

Figure S2. Perceived feasibility of treatment items in a health centre setting by healthcare practitioner panel.

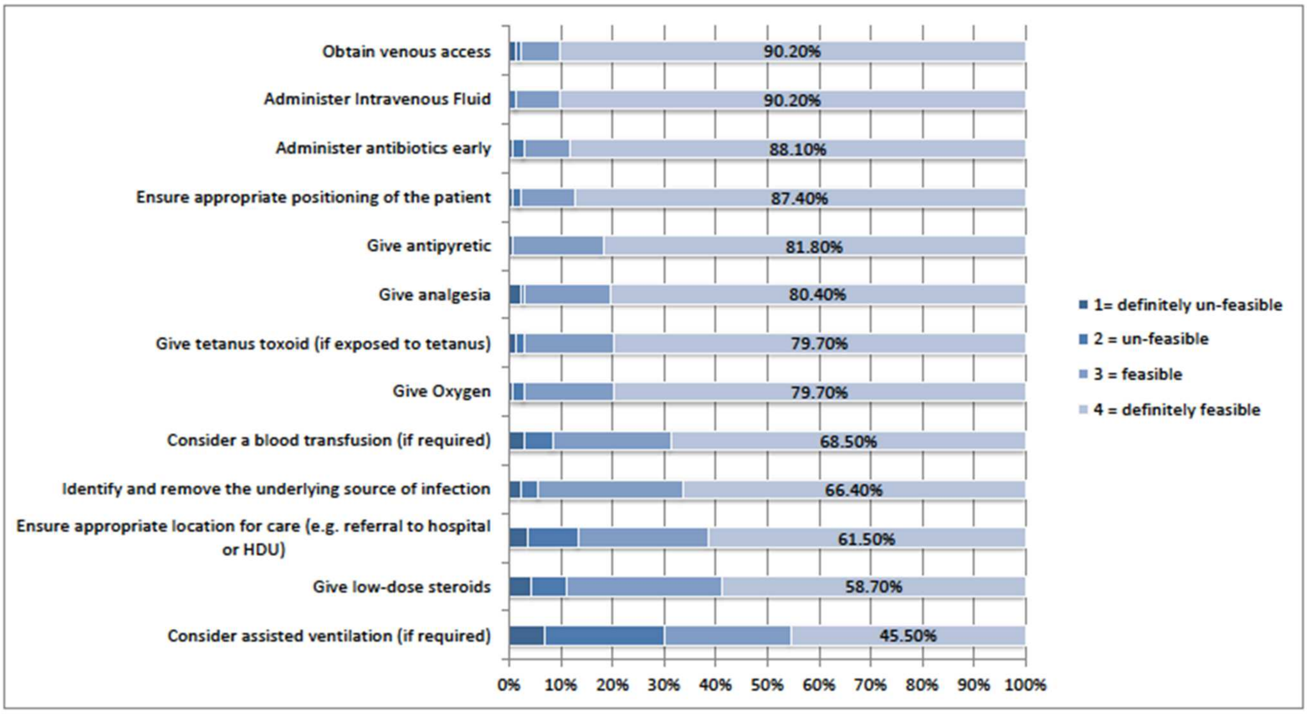

Figure S3. Perceived feasibility of treatment items in a hospital setting by healthcare practitioner panel.

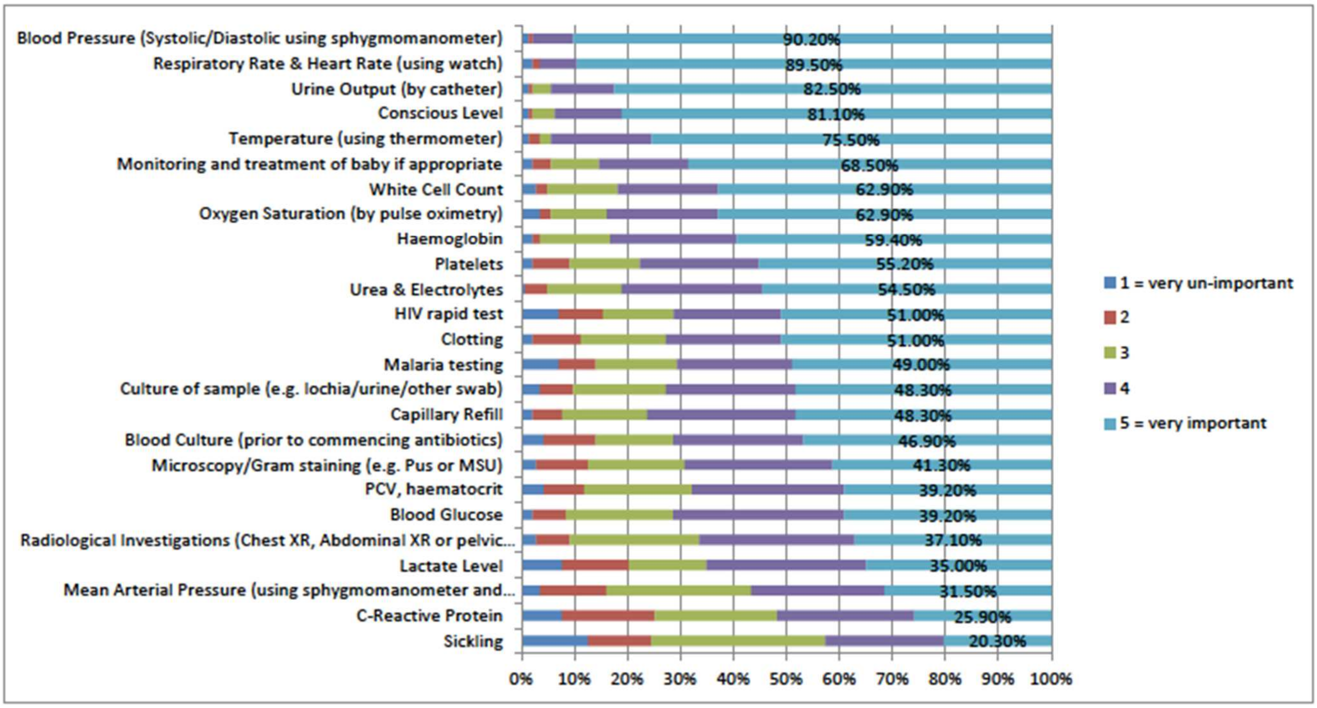

Figure S4. Perceived importance of monitoring items by healthcare practitioner panel.

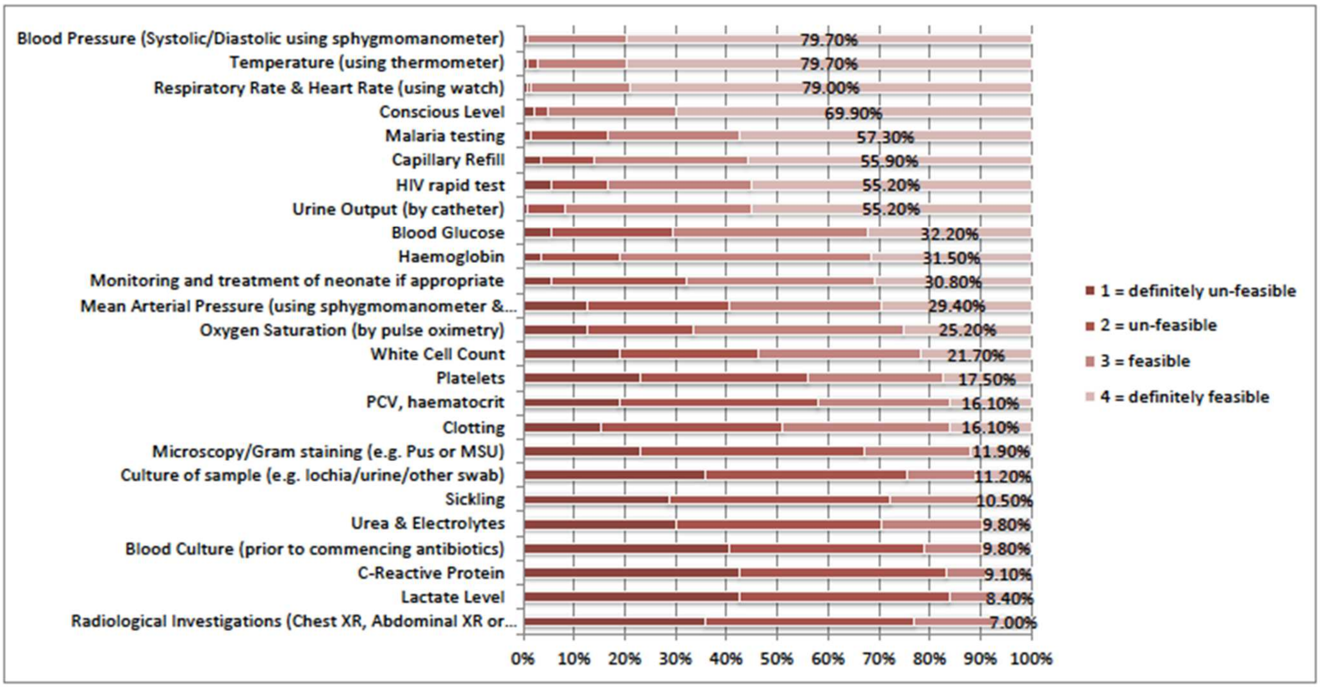

Figure S5. Perceived feasibility of monitoring items in a health centre setting by healthcare practitioner panel.

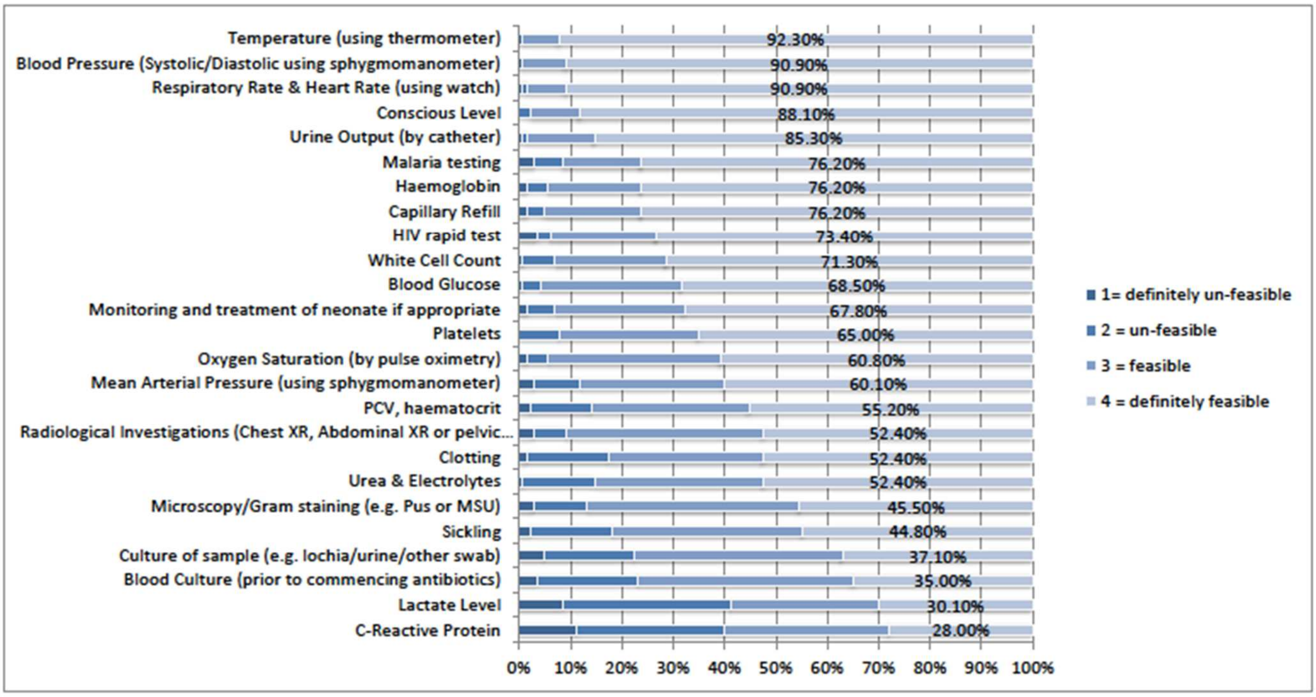

Figure S6. Perceived feasibility of monitoring items in a hospital setting by healthcare practitioner panel.

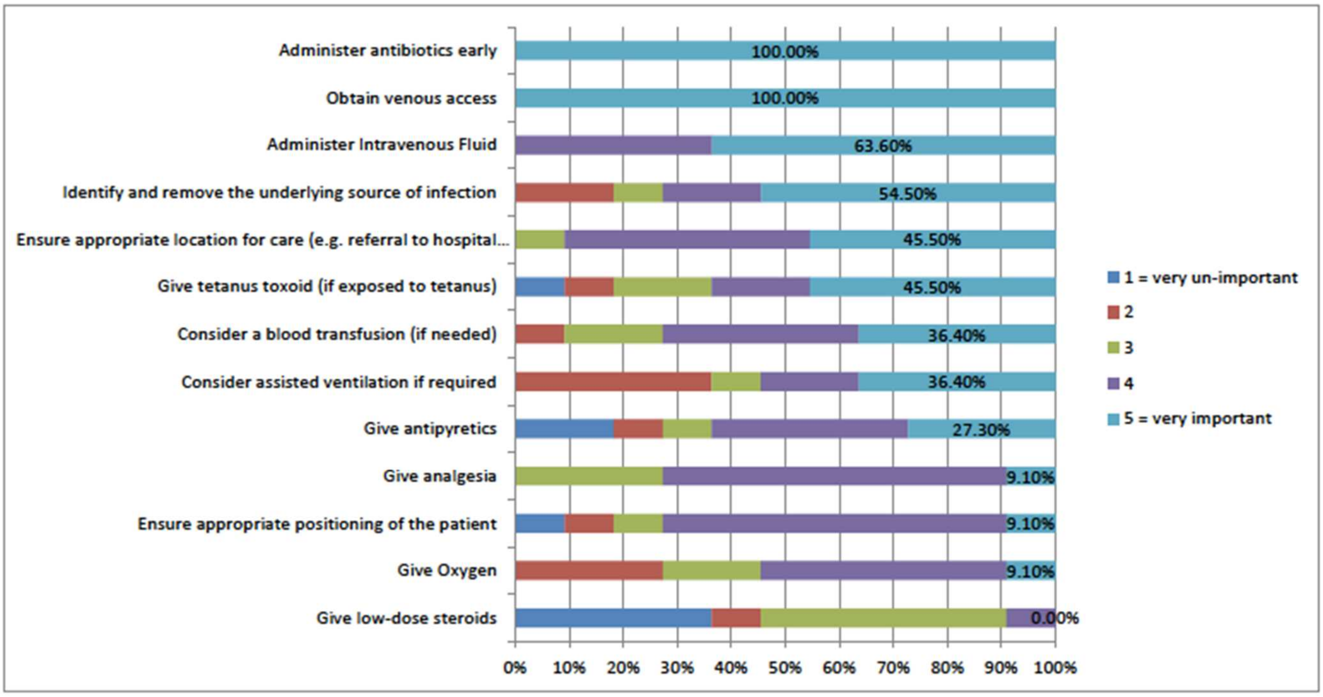

Figure S7. Perceived importance of treatment items by expert panel.

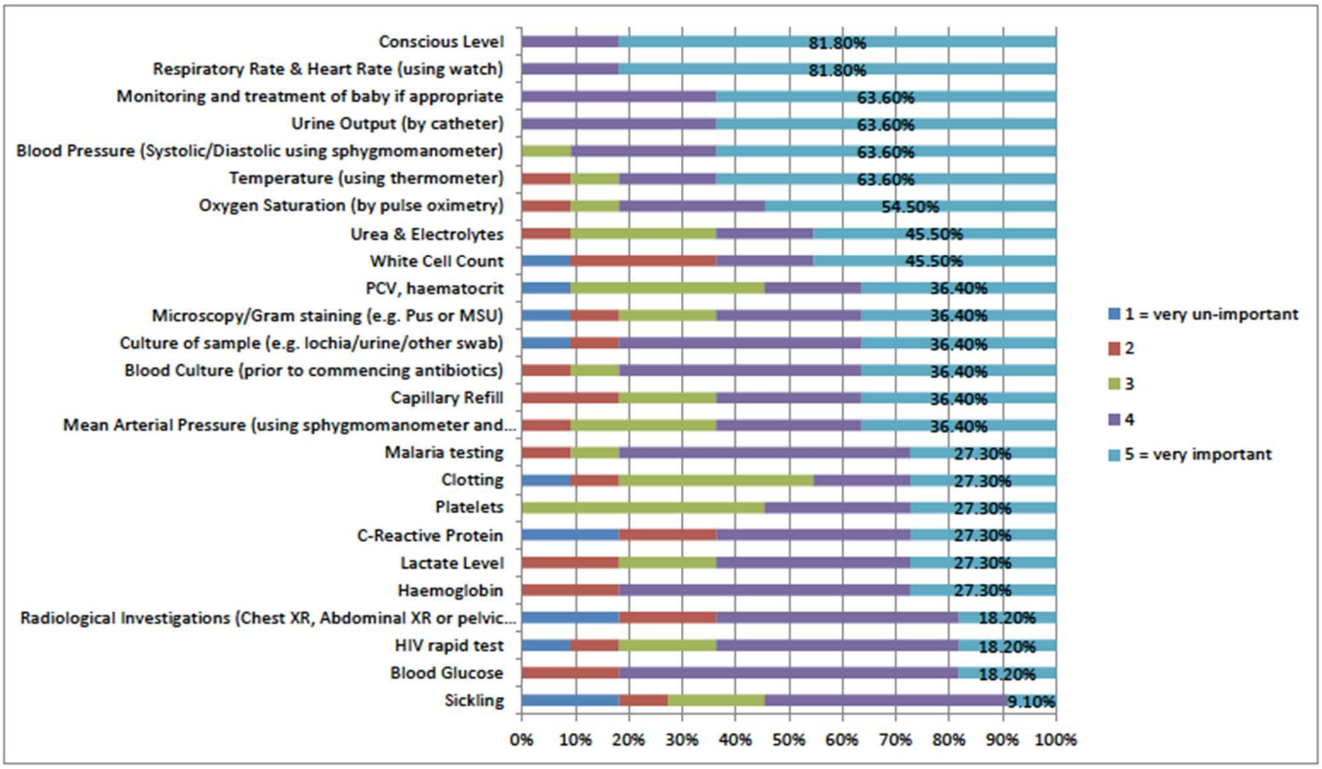

Figure S8. Perceived importance of monitoring items by expert panel.
