# Supplementary material for: Development of the FAST‐M maternal sepsis bundle for use in low‐resource settings: a modified Delphi process
Source: BJOG. 2019 Dec 5;127(3):416–23. doi: 10.1111/1471-0528.16005 (PMC7384197; doi:10.1111/1471-0528.16005)
Supplement: Supplementary file 2 — Appendix S1. List of international guidance, national guidelines and original articles used to identify treatment and monitoring items for inclusion in the modified Delphi process. Appendix S2. Questionnaire used in rounds one and two of the Delphi study. Appendix S3. Questionnaire used in round three of the Delphi study. [file BJO-127-416-s002.pdf]

Appendix S1. List of international guidance, national guidelines and original articles used to identify treatment and monitoring items for inclusion in the modified Delphi process.

National and international guidelines as well as relevant articles within the literature were reviewed by FT and DL until saturation was achieved.

International guidance

- World Health Organization. Integrated Management of Pregnancy And Childbirth. Managing complications in pregnancy and childbirth: a guide for midwives and doctors. World Health Organization, Geneva, 2003
- World Health Organization. Education material for teachers of midwifery: midwifery education modules, 2nd edition. World Health Organization, Geneva, 2008
- World Health Organization. IMAI District Clinician Manual. Hospital Care for Adolescents and Adults: Guidelines for the management of common illnesses with limited resource, volume 2. World Health Organization, Geneva, 2011
- World Health Organization: WHO Model List of Essential Medicines, 16th edition. http://www.who.int/medicines/publications/essentialmedicines/Updated_sixteenth_adult_list_en.pdf (accessed May 2011).
- Medicines Sans Frontieres. Clinical guidelines - Diagnosis and treatment manual. 2013 Edition.
- Dellinger RP, Levy MM, Rhodes A, et al: Surviving Sepsis Campaign: International guidelines for management of severe sepsis and septic shock. Intensive Care Med 2013; 39(2):165-228 and Crit Care Med 2013; 41(2):580- 637.
- Levy MM, Fink MP, Marshall JC et al. 2001 SCCM/ESICM/ACCP/ATS/SIS International Sepsis Definitions Conference. Intensive Care Med 29(4):530-538.
- Marik PE, Pastores SM, Annane D, et al. Recommendations for the diagnosis and management of corticosteroid insufficiency in critically ill adult patients: consensus statements from an international task force by the American College of Critical Care Medicine. Crit Care Med 36(6):1937–1949.
- Collins S, Arulkumaran S, Hayes K, Jackson S, Impey L. Oxford Handbook of Obstetrics and Gynaecology, 2nd Edition. Oxford University Press, Oxford, 2008.
- Van Der Spuy ZM and Anthony J. Handbook of Obstetrics and Gynaecology. Oxford University Press Southern Africa, 2003.

National Guidelines

- Ministry of Health and Social Welfare, The United Republic of Tanzania. Standard Treatment Guidelines and Essential Medicines List, 4th Edition, 2013
- Food, Medicine And Health Care Administration And Control Authority of Ethiopia. Standard Treatment Guidelines For General Hospitals 3rd Edition, 2014
- Ministry of Health, Ghana. Standard Treatment Guidelines, 6th Edition, 2010
- Ministry of Health, Malawi. Malawi Standard Treatment Guidelines, 5th Edition, 2015
- Ministry of Medical Services and Ministry of Public Helath & Sanitation, Republic of Kenya. Clinical Management and Referral Guidelines – Volume III: Clinical Guidelines for Management and Referral of Common Conditions at Levels 4-6: Hospitals, 2009

- Ministry of Health, Uganda. Uganda Clinical Guidelines 2012; National Guidelines for Management of Common Conditions, 2012
- The UK Sepsis Trust. Screening and Action Tool for Use in Pregnancy. Accessed from: https://sepsistrust.org/professional-resources/clinical/
- Maternal Health Division, Ministry of Health & Family Welfare, Government of India. Trainers' guide for training of Medical Officers in Pregnancy Care and Management of Common Obstetric Complications, 2009
- Health & Family Welfare Department, Government of Odisha. Standard Treatment Guideline & Essential Medicine List (For Pregnant Women), 2012
- The Medical Microbiology & Infectious Diseases Society of Pakistan: Guidelines for the Adult Patient for Initial Management of Sepsis/Severe Sepsis/Septic Shock: 2015
- Ministry of Health and Population, Government of Nepal. National Antibiotic Treatment Guidelines, 2014
- Family Health Division, Ministry of Health, Nepal. National Medical Standard for Reproductive Health Volume II, 2003
- Royal College of Obstetricians and Gynaecologists. Bacterial Sepsis in Pregnancy. Green-top Guideline No.64a, 2012
- Royal College of Obstetricians and Gynaecologists. Bacterial Sepsis in Pregnancy. Green-top Guideline No.64b, 2012

Original Articles

- Dünser MW, Festic E, Dondorp A et al. Recommendations for sepsis management in resource-limited settings. Intensive Care Med. 2012;38(4):557-74. doi: 10.1007/s00134-012-2468-5.
- Cheng AC, West TE, Limmathurotsakul D, Peacock SJ. Strategies to Reduce Mortality from Bacterial Sepsis in Adults in Developing Countries. PLoS Med. 2008;5(8):e175. doi: 10.1371/journal.pmed.0050175.
- Martin-Loeches I, Levy MM, Artigas A. Management of severe sepsis: advances, challenges, and current status. Drug Des Devel Ther. 2015;9:2079-88. doi: 10.2147/DDDT.S78757.
- van Dillen J, Zwart J, Schutte J, van Roosmalen J. Maternal sepsis: epidemiology, etiology and outcome. Curr Opin Infect Dis. 2010;23(3):249-54. doi: 10.1097/QCO.0b013e328339257c.
- Barton JR, Sibai BM. Severe sepsis and septic shock in pregnancy. Obstet Gynecol. 2012;120(3):689-706. doi: 10.1097/AOG.0b013e318263a52d.
- Becker JU, Theodosis C, Jacob ST, Wira CR, Groce NE. Surviving sepsis in low-income and middle-income countries: new directions for care and research. Lancet Infect Dis. 2009;9(9):577-82. doi: 10.1016/S1473-3099(09)70135-5.
- Arulkumaran N, Singer M. Puerperal sepsis. Best Pract Res Clin Obstet Gynaecol. 2013;27(6):893-902. doi: 10.1016/j.bpobgyn.2013.07.004.
- Sriskandan S. Severe peripartum sepsis. J R Coll Physicians Edinb. 2011;41(4): 339-46. doi: 10.4997/JRCPE.2011.411.
- Dünser MW, Takala J, Ulmer H, et al. Arterial blood pressure during early sepsis and outcome. Intensive Care Med. 2009;35(7):1225-33. doi: 10.1007/s00134-009-1427-2.

- Hollenberg SM, Ahrens TS, Annane D, et al. Practice parameters for hemodynamic support of sepsis in adult patients: 2004 update. Crit Care Med. 2004;32(9):1928-48.
- Hurtado FJ, Nin N. The role of bundles in sepsis care. Crit care clin. 2006;22(3):521–529.
- Weippl G. Infectious toxic hypotension--effect and dosage of midodrine. Padiatr Padol. 1979;14(2):211-6.
- Rivers E, Nguyen B, Havstad S, et al. Early goal-directed therapy in the treatment of severe sepsis and septic shock. N Engl J Med. 2001;345(19):1368-77.
- Zanotti Cavazzoni SL, Dellinger RP. Hemodynamic optimization of sepsis-induced tissue hypoperfusion. Crit Care. 2006;10 Suppl 3:S2.
- Mock C, Visser L, Denno D, Maier R. Aggressive fluid resuscitation and broad spectrum antibiotics decrease mortality from typhoid ileal perforation. Trop Doct. 1995;25(3):115-7.
- Jacob ST, Moore CC, Banura P, et al. Severe sepsis in two Ugandan hospitals: a prospective observational study of management and outcomes in a predominantly HIV-1 infected population. PLoS One. 2009 Nov;4(11):e7782. doi: 10.1371/journal.pone.0007782.
- Upadhyay M, Singhi S, Murlidharan J, Kaur N, Majumdar S. Randomized evaluation of fluid resuscitation with crystalloid (saline) and colloid (polymer from degraded gelatin in saline) in pediatric septic shock. Indian Pediatr. 2005;42(3):223-31.
- Perel P, Roberts I, Ker K. Colloids versus crystalloids for fluid resuscitation in critically ill patients. Cochrane Database Syst Rev. 2013;(2):CD000567. doi: 10.1002/14651858.CD000567.pub6.
- Akech S, Ledermann H, Maitland K. Choice of fluids for resuscitation in children with severe infection and shock: systematic review. BMJ. 2010;341:c4416. doi: 10.1136/bmj.c4416.
- Annane D, Bellissant E, Bollaert PE, et al. Corticosteroids in the treatment of severe sepsis and septic shock in adults: a systematic review. JAMA. 2009;301(22):2362-75. doi: 10.1001/jama.2009.815.
- Sligl WI, Milner DA Jr, Sundar S, Mphatswe W, Majumdar SR. Safety and efficacy of corticosteroids for the treatment of septic shock: A systematic review and meta-analysis. Clin Infect Dis. 2009;49(1):93-101. doi: 10.1086/599343.
- Annane D, Vignon P, Renault A, et al. Norepinephrine plus dobutamine versus epinephrine alone for management of septic shock: a randomised trial. Lancet. 2007;370(9588):676-84.
- Myburgh JA, Higgins A, Jovanovska A, et al. A comparison of epinephrine and norepinephrine in critically ill patients. Intensive Care Med. 2008;34:2226–2234.
- De Backer D, Biston P, Devriendt J, et al. Comparison of dopamine and norepinephrine in the treatment of shock. N Engl J Med. 2010;362(9):779–789
- Moran JL, Graham PL, Rockliff S, Bersten AD. Updating the evidence for the role of corticosteroids in severe sepsis and septic shock: a Bayesian meta-analytic perspective. Crit Care. 2010;14(4):R134. doi: 10.1186/cc9182.
- Bernard GR, Luce JM, Sprung CLet al. High-dose corticosteroids in patients with the adult respiratory distress syndrome. N Engl J Med. 1987;317(25):1565–1570.

- Walker IA, Merry AF, Wilson IH, et al. Global oximetry: an international anaesthesia quality improvement project. Anaesthesia. 2009;64(10):1051–1060. doi: 10.1111/j.1365-2044.2009.06067.x.
- Duke T, Subhi R, Peel D, Frey B. Pulse oximetry: technology to reduce child mortality in developing countries. Ann Trop Paediatr. 2009;29(3):165–175. doi: 10.1179/027249309X12467994190011.
- Duke T, Wandi F, Jonathan M, et al. Improved oxygen systems for childhood pneumonia: a multihospital effectiveness study in Papua New Guinea. Lancet. 2008;372(9646):1328–1333. doi: 10.1016/S0140-6736(08)61164-2.
- Tantipong H, Morkchareonpong C, Jaiyindee S, Thamlikitkul. Randomized controlled trial and metaanalysis of oral decontamination with 2% chlorhexidine solution for the prevention of ventilator-associated pneumonia. Infect Control Hosp Epidemiol. 2008;29(2):131–136. doi: 10.1086/526438.
- Drakulovic MB, Torres A, Bauer TT, et al. Supine body position as a risk factor for nosocomial pneumonia in mechanically ventilated patients: a randomised trial. Lancet. 1999;354(9193):1851–1858.
- Torres A, Serra-Batlles J, Ros E, et al. Pulmonary aspiration of gastric contents in patients receiving mechanical ventilation: the effect of body position. Ann Intern Med. 1992;116(7):540–543.
- Duke T, Graham SM, Cherian MN, et al. Oxygen is an essential medicine: a call for international action. Int J Tuberc Lung Dis. 2010;14(11):1362-1368.
- Hussain SF, Haqqee R, Iqbal J. Non-invasive ventilation in the management of acute respiratory failure in Pakistan. Trop Doct. 2004;34(4):238–239.
- George IA, John G, John P, Peter JV, Christopher S. An evaluation of the role of noninvasive positive pressure ventilation in the management of acute respiratory failure in a developing country. Indian J Med Sci. 2007;61(9):495–504.
- Han YY, Carcillo JA, Dragotta MA, et al. Early reversal of pediatric-neonatal septic shock by community physicians is associated with improved outcome. Pediatrics 2003;112(4):793–799.
- Oliveira CF, Nogueira de Sa´ FR, Oliveira DS, et al. Time- and fluid sensitive resuscitation for hemodynamic support of children in septic shock: barriers to the implementation of the American College of Critical Care Medicine/Pediatric Advanced Life Support Guidelines in a pediatric intensive care unit in a developing world. Pediatr Emerg Care. 2008;24(12):810–815. doi: 10.1097/PEC.0b013e31818e9f3a.
- Kumar A, Roberts D, Wood KE, et al. Duration of hypotension before initiation of effective antimicrobial therapy is the critical determinant of survival in human septic shock. Crit Care Med. 2006;34(6):1589–1596.
- Kumar A, Ellis P, Arabi Y, et al. Initiation of inappropriate antimicrobial therapy results in a fivefold reduction of survival in human septic shock. Chest. 2009;136(5):1237–1248. doi: 10.1378/chest.09-0087.
- Gaieski DF, Mikkelsen ME, Band RA, et al. Impact of time to antibiotics on survival in patients with severe sepsis or septic shock in whom early goal-directed therapy was initiated in the emergency department. Crit Care Med. 2010;38(4):1045–1053. doi: 10.1097/CCM.0b013e3181cc4824.

- Gomes MF, Faiz MA, Gyapong JO, et al. Pre-referral rectal artesunate to prevent death and disability in severe malaria: a placebo-controlled trial. Lancet 2009;373(9663):557–566. doi: 10.1016/S0140-6736(08)61734-1.
- Leibovici L, Shraga I, Drucker M, et al. The benefit of appropriate empirical antibiotic treatment in patients with bloodstream infections. J intern med 1998;244(5):379-86.
- Ibrahim EH, Sherman G, Ward S, Fraser VJ, Kollef MH. The influence of inadequate antimicrobial treatment of bloodstream infections on patient outcomes in the ICU setting. Chest. 2000;118(1):146-55.
- Bochud PY, Bonten M, Marchetti O, Calandra T. Antimicrobial therapy for patients with severe sepsis and septic shock: an evidence-based review. Crit Care Med. 2004;32(11 Suppl):S495-512.
- Shafazand S, Weinacker AB. Blood cultures in the critical care unit: improving utilization and yield. Chest. 2002;122(5):1727–1736.
- Trzeciak S, Dellinger RP, Chansky ME, et al. Serum lactate as a predictor of mortality in patients with infection. Intensive Care Med. 2007;33(6):970-7.
- Sapin V, Nicolet L, Aublet-Cuvelier B, et al. Rapid decrease in plasma D-lactate as an early potential predictor of diminished 28 day mortality. Clin Chem Lab Med. 2006;44(4):492-96.
- Nickerson EK, Wuthiekanun V, Wongsuvan G, et al. Factors predicting and reducing mortality in patients with invasive Staphylococcus aureus disease in a developing country. PLoS One. 2009;4(8):e6512. doi: 10.1371/journal.pone.0006512.
- Rosenthal VD, Maki DG, Mehta A, et al. International Nosocomial Infection Control Consortium report, data summary for 2002-2007, issued January 2008. Am J Infect Control. 2008;36(9):627-37. doi: 10.1016/j.ajic.2008.03.003.
- Rosenthal VD, Maki DG, Rodrigues C, et al. Impact of International Nosocomial Infection Control Consortium (INICC) strategy on central line-associated bloodstream infection rates in the intensive care units of 15 developing countries. Infect Control Hosp Epidemiol. 2010;31(12):1264-72. doi: 10.1086/657140.
- Michard F, Boussat S, Chemla D, et al. Relation between respiratory changes in arterial pulse pressure and fluid responsiveness in septic patients with acute circulatory failure. Am J Respir Crit Care Med. 2000;162(1):134-8.
- Moore CC, Jacob ST, Pinkerton R, et al. Point-of-care lactate testing predicts mortality of severe sepsis in a predominantly HIV type 1-infected patient population in Uganda. Clin Infect Dis. 2008;46(2):215-22. doi: 10.1086/524665.
- Baelani I, Jochberger S, Laimer T, et al. Availability of critical care resources to treat patients with severe sepsis or septic shock in Africa: a self-reported, continent-wide survey of anaesthesia providers. Crit Care. 2011;15(1):R10.
- Bataar O, Lundeg G, Tsenddorj G, et al. Nationwide survey on resource availability for implementing current sepsis guidelines in Mongolia. Bull World Health Organ. 2010;88(11):839-846.
- Gardner SL. Sepsis in the neonate. Crit Care Nurs Clin North Am. 2009;21(1):121-41, vii. doi: 10.1016/j.ccell.2008.11.002.
- Brunkhorst FM, Engel C, Bloos F, et al. Intensive insulin therapy and pentastarch resuscitation in severe sepsis. N Engl J Med. 2008;10;358(2):125-39. doi: 10.1056/NEJMoa070716.

- Preiser JC, Devos P. Clinical experience with tight glucose control by intensive insulin therapy. Crit Care Med. 2007;35(9 Suppl):S503-7.
- Masterton RG. Antibiotic de-escalation. Crit Care Clin. 2011;27(1):149-62. doi: 10.1016/j.ccc.2010.09.009.
- Mentzelopoulos SD, Pratikaki M, Platsouka E, et al. Prolonged use of carbapenems and colistin predisposes to ventilator-associated pneumonia by pandrug-resistant Pseudomonas aeruginosa. Intensive Care Med. 2007;33(9):1524-32.
- Rattanataweeboon P, Vilaichone W,Vannasaeng S. Stress hyperglycemia in patients with sepsis. J Med Assoc Thai. 2009;92(Suppl 2):S88–S94.
- Ssekitoleko R, Jacob ST, Banura P, et al. Hypoglycemia at admission is associated with inhospital mortality in Ugandan patients with severe sepsis. Crit Care Med. 2011;39(10):2271-6. doi: 10.1097/CCM.0b013e3182227bd2.
- Osime U, Lawrie J, Lawrie H. Post-operative deep vein thrombosis incidence in Nigerians. Niger Med J. 1976;6(1):26–28.
- Colin JF, Rottcher KH, Dhali DP. Letter: venous thrombosis in Africans. Lancet. 1975; 1(7903):408.
- Lee AD, Stephen E, Agarwal S, Premkumar P. Venous thromboembolism in India. Eur J Vasc Endovasc Surg 2009;37(4):482–485.
- Sotunmbi PT, Idowu AT, Akang EE,Aken'Ova YA. Prevalence of venous thromboembolism at postmortem in an African population: a cause for concern. Afr J Med Med Sci 2006;35(3):345–348.
- Samama MM, Cohen AT, Darmon JY, et al. A comparison of enoxaparin with placebo for the prevention of venous thromboembolism in acutely ill medical patients. Prophylaxis in Medical Patients with Enoxaparin Study Group. N Engl J Med 1999;341(11):793–800.
- Amaragiri SV, Lees TA. Elastic compression stockings for prevention of deep vein thrombosis. Cochrane Database Syst Rev 2000;(3):CD001484.
- Doig GS, Heighes PT, Simpson F, Sweetman EA, Davies AR. Early enteral nutrition, provided within 24 h of injury or intensive care unit admission, significantly reduces mortality in critically ill patients: a meta-analysis of randomised controlled trials. Intensive Care Med 2009;35(12):2018–2027. doi: 10.1007/s00134-009-1664-4
- Berkenstadt H, Segal E, Mayan H, et al. The pharmacokinetics of morphine and lidocaine in critically ill patients. Intensive Care Med. 1999;25(1):110-2.
- Milbrandt EB, Kersten A, Kong L, et al. Haloperidol use is associated with lower hospital mortality in mechanically ventilated patients. Crit Care Med 2005;33(1):226–229; discussion 263-5.
- Reader TW, Flin R, Mearns K, Cuthbertson BH. Developing a team performance framework for the intensive care unit. Crit Care Med 2009;37(5):1787-1793. doi: 10.1097/CCM.0b013e31819f0451.
- Ottestad E, Boulet JR, Lighthall GK. Evaluating the management of septic shock using patient simulation. Crit Care Med 2007;35(3):769-775.
- Pronovost P, Berenholtz S, Dorman T, et al. Improving communication in the ICU using daily goals. J Crit Care 2003;18(2):71-75.
- Sprung CL, Sakr Y, Vincent JL, et al. An evaluation of systemic inflammatory response syndrome signs in the Sepsis Occurrence In Acutely Ill Patients (SOAP) study. Intensive Care Med 2006;32(3): 421-427.

- Lima A, Jansen TC, van Bommel J, Ince C, Bakker J. The prognostic value of the subjective assessment of peripheral perfusion in critically ill patients. Crit Care Med 2009;37(3):934-938. doi: 10.1097/CCM.0b013e31819869db.
- Jansen TC, van Bommel J, Mulder PG, et al. Prognostic value of blood lactate levels: does the clinical diagnosis at admission matter? J Trauma 2009;66(2):377-385. doi: 10.1097/TA.0b013e3181648e2f.

Appendix S2. Questionnaire used in rounds one and two of the Delphi study.

1. Which country do you mainly work in? ________________________________

2. What is your job title? ________________________________

3. What setting do you work in? ________________________________

4. In the last six months, have you managed a patient with maternal sepsis? ____________

5. In your opinion, how **important** are the following items in the initial **treatment** of severe maternal sepsis (e.g. to be initiate within 3 hours) in low and low-middle income countries?

Please only think about the importance of each item. Feasibility will be asked about later in the survey.

Please rate the following:

	1 = very un-important	2	3	4	5 = very important
**Administer intravenous fluid**					
**Obtain venous access**					
**Administer antibiotics early**					
**Give oxygen**					
**Consider assisted ventilation if required**					
**Ensure appropriate positioning of patient**					
**Identify and remove the underlying source of infection**					
**Give analgesia**					
**Give tetanus toxoid** (if exposed to tetanus)					
**Give antipyretics**					
**Consider a blood transfusion** (if needed)					
**Give low-dose steroids**					
**Ensure appropriate locate or care** (e.g. referral to hospital or HDU)					

6. Do you think any items are currently **missing** from the previous set of questions? If so please comment below:

---

7. In your opinion, how **important** are the following items in **monitoring** patients already identified as having severe maternal sepsis (within first three hours), in low and low-middle income countries?

Please rate the following:

	1 = very un-important	2	3	4	5 = very important
**Respiratory rate and heart rate** (using a watch)					
**Temperature** (using thermometer)					

**Blood pressure** (Systolic/Diastolic using sphygmomanometer)					
**Mean arterial pressure** (using sphygmomanometer and calculator)					
**Conscious level**					
**Urine output** (by catheter)					
**Capillary refill**					
**Oxygen saturations** (by pulse oximetry)					
**Blood glucose**					
**Blood culture** (prior to commencing antibiotics)					
**Culture of sample** (e.g. lochia/urine/other swab)					
**Microscopy/Gram staining** (e.g. Pus or MSU)					
**Haemoglobin**					
**Lactate level**					
**C-Reactive Protein**					
**White Cell Count**					
**Platelets**					
**Urea & Electrolytes**					
**Clotting**					
**PCV, haematocrit**					
**Sickling**					
**HIV rapid test**					
**Malaria testing**					
**Radiological investigations** (Chest XR, Abdominal XR or pelvic USS)					
**Monitoring and treatment of baby** (if appropriate)					

8. Do you think any items are currently **missing** from the previous set of questions? If so please comment below:

---

9. How **feasible** do you think the use of the following will be in a **health centre** in a low and low-middle income country?

(By health centre we are considering a health facility which is aiming to provide basic emergency obstetric care. As such this is a facility where vaginal deliveries will usually take place, as well as routine antenatal and post-natal care but there is no recourse to caesarean section. There may be non- physician clinicians present as well as nurses and midwives; however it is unlikely there will be any doctors working at this sort of facility.)

Please rate the following:

	1 = definitely un-feasible	2 = un-feasible	3 = feasible	4 = definitely feasible
**Administer intravenous fluid**				
**Obtain venous access**				
**Administer antibiotics early**				
**Give oxygen**				

**Consider assisted ventilation if required**				
**Ensure appropriate positioning of patient**				
**Identify and remove the underlying source of infection**				
**Give analgesia**				
**Give tetanus toxoid** (if exposed to tetanus)				
**Give antipyretics**				
**Consider a blood transfusion** (if needed)				
**Give low-dose steroids**				
**Ensure appropriate locate or care** (e.g. referral to hospital or HDU)				
**Respiratory rate and heart rate** (using a watch)				
**Temperature** (using thermometer)				
**Blood pressure** (Systolic/Diastolic using sphygmomanometer)				
**Mean arterial pressure** (using sphygmomanometer and calculator)				
**Conscious level**				
**Urine output** (by catheter)				
**Capillary refill**				
**Oxygen saturations** (by pulse oximetry)				
**Blood glucose**				
**Blood culture** (prior to commencing antibiotics)				
**Culture of sample** (e.g. lochia/urine/other swab)				
**Microscopy/Gram staining** (e.g. Pus or MSU)				
**Haemoglobin**				
**Lactate level**				
**C-Reactive Protein**				
**White Cell Count**				
**Platelets**				
**Urea & Electrolytes**				
**Clotting**				
**PCV, haematocrit**				
**Sickling**				
**HIV rapid test**				
**Malaria testing**				
**Radiological investigations** (Chest XR, Abdominal XR or pelvic USS)				
**Monitoring and treatment of baby** (if appropriate)				

10. If you have any additional comments please provide them below:

11. How **feasible** do you think the use of the following will be in a **district hospital** (or any facility where comprehensive essential obstetric care is available) in a low and low-middle income country?

Please rate the following:

	1 = definitely un-feasible	2 = un-feasible	3 = feasible	4 = definitely feasible
**Administer intravenous fluid**				
**Obtain venous access**				
**Administer antibiotics early**				
**Give oxygen**				
**Consider assisted ventilation if required**				
**Ensure appropriate positioning of patient**				
**Identify and remove the underlying source of infection**				
**Give analgesia**				
**Give tetanus toxoid** (if exposed to tetanus)				
**Give antipyretics**				
**Consider a blood transfusion** (if needed)				
**Give low-dose steroids**				
**Ensure appropriate locate or care** (e.g. referral to hospital or HDU)				
**Respiratory rate and heart rate** (using a watch)				
**Temperature** (using thermometer)				
**Blood pressure** (Systolic/Diastolic using sphygmomanometer)				
**Mean arterial pressure** (using sphygmomanometer and calculator)				
**Conscious level**				
**Urine output** (by catheter)				
**Capillary refill**				
**Oxygen saturations** (by pulse oximetry)				
**Blood glucose**				
**Blood culture** (prior to commencing antibiotics)				
**Culture of sample** (e.g. lochia/urine/other swab)				
**Microscopy/Gram staining** (e.g. Pus or MSU)				
**Haemoglobin**				
**Lactate level**				
**C-Reactive Protein**				
**White Cell Count**				
**Platelets**				
**Urea & Electrolytes**				

**Clotting**				
**PCV, haematocrit**				
**Sickling**				
**HIV rapid test**				
**Malaria testing**				
**Radiological investigations** (Chest XR, Abdominal XR or pelvic USS)				
**Monitoring and treatment of baby** (if appropriate)				

12. If you have any additional comments, please provide them below:

Thank you very much for completing this survey.

This survey will form the first stage of the Delphi process that will help us design the bundle. We would like to contact you again with a refined set of questions to reach consensus on the right components of the bundle.

If you are happy to be contacted again please enter your email address below, we will only contact you up to two more times as part of this process.

____

Appendix S3. Questionnaire used in round three of the Delphi study.

Following completion of the first and second rounds of the Delphi, we have arranged the top ranking elements in the below tables, applying a rank according to the mean score given for the importance for each element, as identified by both panels.

The steering committee have reviewed the responses for both practitioner and expert panels and found there was consensus on the five most important **treatment** elements.

The five most important **treatment** elements (and mean score) using results from both panels

Practitioner Panel	Rank	Expert Panel
**Antibiotics**<br>Mean = 4.90, SD 0.58	**1**	**Antibiotics**<br>Mean = 5.00, SD 0.00
**IV Access***<br>Mean = 4.83, SD 0.69	**2**	**IV Access***<br>Mean = 5.00, SD 0.00
**IV Fluids**<br>Mean = 4.71, SD 0.76	**3**	**IV Fluids**<br>Mean = 4.64, SD 0.48
**Source Control**<br>Mean = 4.56, SD 0.82	**4**	**Location**<br>Mean = 4.36, SD 0.34
**Location**<br>Mean = 4.39, SD 0.97	**5**	**Source Control**<br>Mean = 4.09, SD 1.16

*IV access will be removed from the final ranking as it is intrinsic to two other top ranked elements (administration of IV fluids and IV antibiotics).

The steering committee also found there was consensus on the following seven most important **monitoring** elements.

The seven most important **monitoring** elements (and mean score) using results from both panels

Practitioner Panel	Rank	Expert Panel
**Blood Pressure**<br>Mean = 4.85, SD 0.58	**1**	**Respiratory Rate & Heart Rate**<br>Mean = 4.82, SD 0.39
**Respiratory Rate & Heart Rate**<br>Mean = 4.80, SD 0.70	**2**	**Conscious Level**<br>Mean = 4.82, SD 0.39
**Urine Output**<br>Mean = 4.73, SD 0.69	**3**	**Urine Output**<br>Mean = 4.64, SD 0.48
**Conscious Level**<br>Mean = 4.71, SD 0.71	**4**	**Monitoring of Baby**<br>Mean = 4.64, SD 0.48
**Temperature**<br>Mean = 4.65, SD 0.75	**5**	**Blood Pressure**<br>Mean = 4.55, SD 0.66
**Monitoring of Baby**<br>Mean = 4.46, SD 0.94	**6**	**Temperature**<br>Mean = 4.27, SD 0.96
**Oxygen Saturation****<br>Mean = 4.38, SD 1.00	**7**	**Oxygen Saturation****<br>Mean = 4.27, SD 0.96

** Oxygen Saturation was ranked highly in terms of importance but many practitioners reported it was not currently feasible in their setting. Following discussions at the WHO meeting in February, it was suggested that due to its importance this should be included, and recommended for use in those facilities where it was

available.

In addition to the tables above, please find below some figures showing further results from the first two rounds.

Figures 1 & 2 are scatter plots showing the importance against feasibility scores to show the trend for the elements in both hospital and health centre settings from round 1.

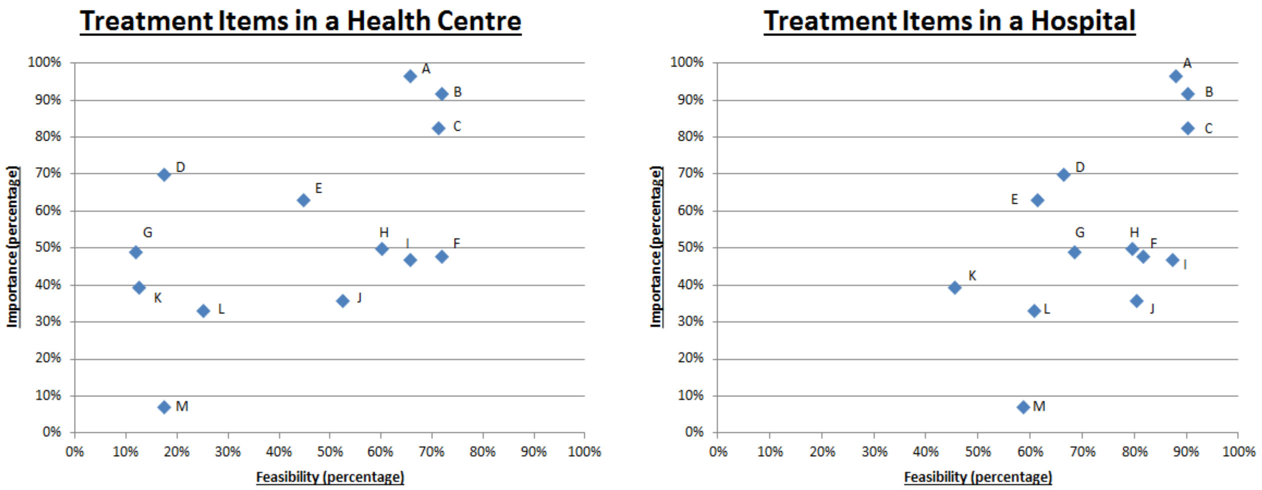

Figure 1: Scatter Plot showing the percentage of respondents who score an item as "definitely feasible" plotted against the percentage scoring the item as "very important" for treatment elements in both a health centre and hospital setting

**A**	Administer antibiotics early	**H**	Give tetanus toxoid (if exposed to tetanus)
**B**	Obtain venous access	**I**	Ensure appropriate positioning of the patient
**C**	Administer Intravenous Fluid	**J**	Give analgesia
**D**	Identify and remove the underlying source of infection	**K**	Consider assisted ventilation if required
**E**	Ensure appropriate location for care (e.g. referral to hospital or HDU)	**L**	Give Oxygen
**F**	Give antipyretics	**M**	Give low-dose steroids
**G**	Consider a blood transfusion (if needed)		

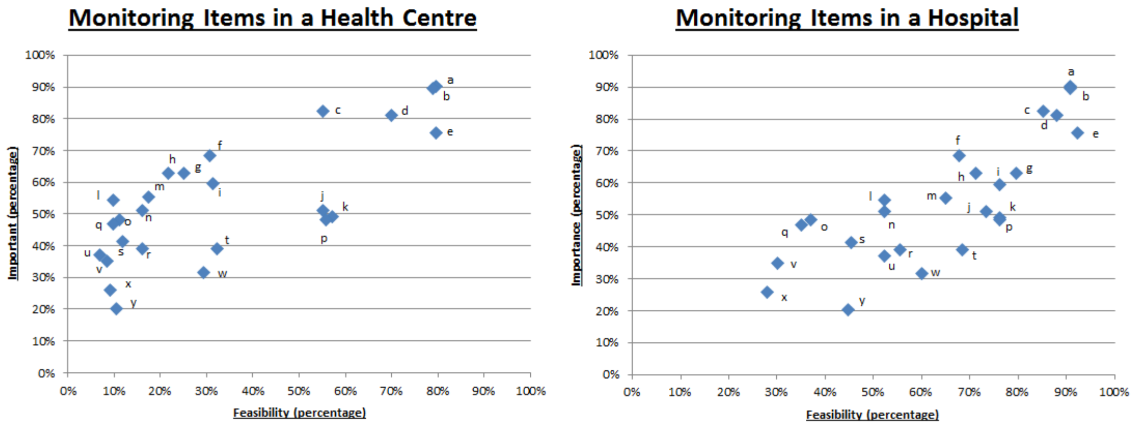

Figure 2: Scatter Plot showing the percentage of respondents who score an item as "definitely feasible" plotted against the percentage scoring the item as "very important" for monitoring elements in both a health centre and hospital setting

**a**	Blood Pressure (using sphygmomanometer)	**j**	HIV rapid test	**s**	Microscopy/Gram staining (e.g. Pus or MSU)
**b**	Respiratory Rate & Heart Rate (using watch)	**k**	Malaria testing	**t**	Blood Glucose
**c**	Urine Output (by catheter)	**l**	Urea & Electrolytes	**u**	Radiological Investigations (e.g. XR or USS)
**d**	Conscious Level	**m**	Platelets	**v**	Lactate Level
**e**	Temperature (using thermometer)	**n**	Clotting	**w**	Mean Arterial Pressure
**f**	Monitoring and treatment of baby if appropriate	**o**	Culture of sample (e.g. lochia/urine)	**x**	C-Reactive Protein
**g**	Oxygen Saturation (by pulse oximetry)	**p**	Capillary Refill	**y**	Sickling
**h**	White Cell Count	**q**	Blood Culture (prior to antibiotics)		
**i**	Haemoglobin	**r**	PCV, haematocrit		

Figures 3 & 4 are stacked bar charts showing the distribution of scores given in terms of importance of each item by our expert panel in round 2.

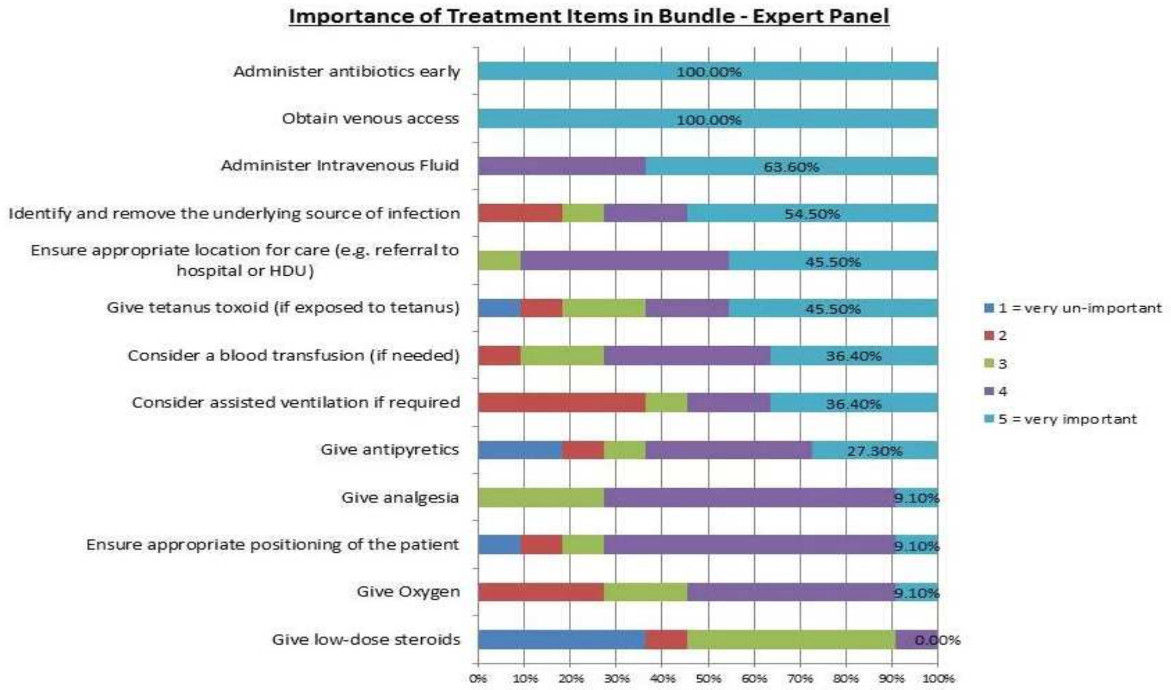

Figure 3: Results from the second round Delphi survey regarding the importance of treatment items using a Likert score out of 5. Data presented as 100% stacked bar chart with colours corresponding to the score given. The percentage highlighted indicates the proportion of respondents giving a score of 5/5 – "very important"

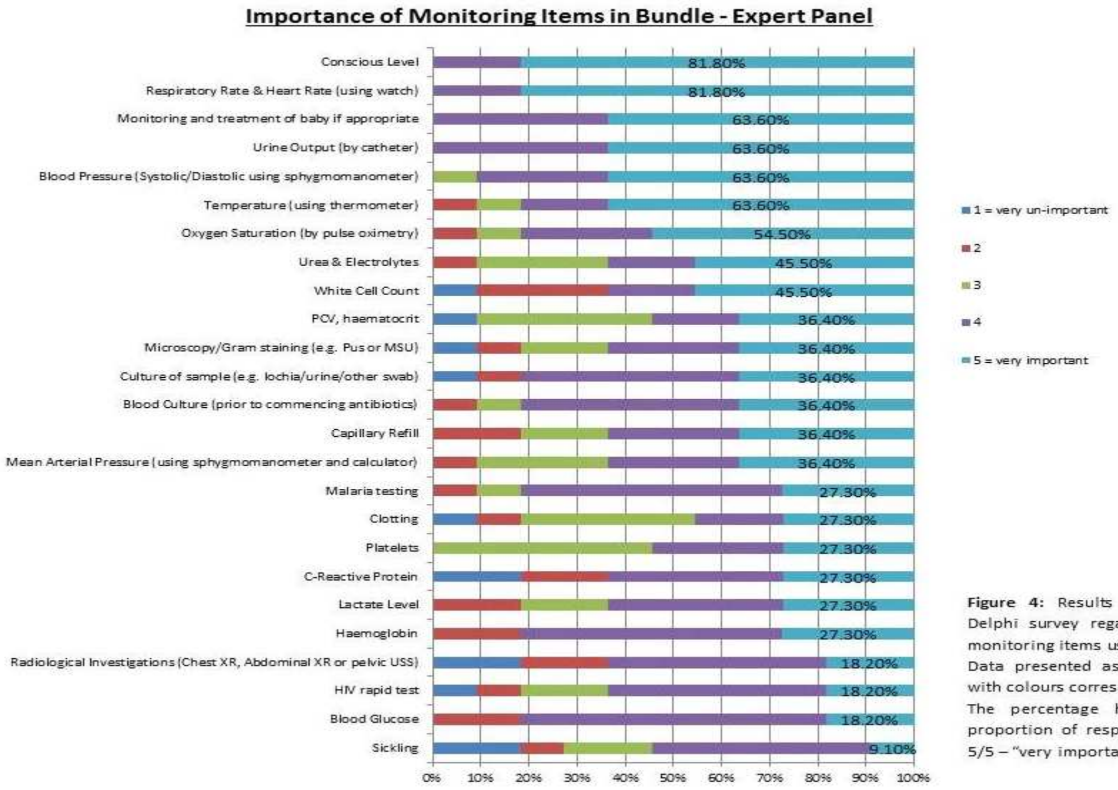

Figure 4: Results from the second round Delphi survey regarding the importance of monitoring items using a Likert score out of 5. Data presented as 100% stacked bar chart with colours corresponding to the score given. The percentage highlighted indicates the proportion of respondents giving a score of 5/5 – "very important"

Considering the above information, the key elements that were consistently found to be both feasible and important are as follows:

- Intravenous fluids
- Intravenous antibiotics
- Source (identification and control)
- Transfer (right facility and level of care)
- Monitoring of mother (blood pressure/ respiratory rate/ heart rate/ conscious level/ temperature/ urine output/ oxygen saturations (if available)) and neonate (if applicable)

1. Do you agree with that the above key elements should be included in the final bundle? Please provide comments if you wish.

Treatment elements YES / NO

Monitoring elements YES / NO

Comments:

2. Are there any treatment or monitoring components you would like to **add** from the final bundle?

3. Are there any treatment or monitoring components you would like to **remove** from the final bundle?

Thank you so much for completing this survey. These results will form the important final part of the Delphi process. We will be sure to contact you again soon to update you regarding the final results.
